# What is the best pulse shape for pacing purposes?

**DOI:** 10.3389/fphys.2025.1480660

**Published:** 2025-03-21

**Authors:** Avinoam Rabinovitch, Doron Braunstein, Ella Smolik, Yaacov Biton, Revital Rabinovitch

**Affiliations:** ^1^ Physics Department, Ben-Gurion University of the Negev, Beer-Sheva, Israel; ^2^ Physics Department, Sami Shamoon College of Engineering, Beer-Sheva, Israel; ^3^ Physics Department, Shamoon College of Engineering, Ashdod Campus, Ashdod, Israel; ^4^ Makif YudAlef, Rishon Lezion, Israel

**Keywords:** nonlinear tissue simulation, electric pulse energy dynamics, FitzHugh-Nagumo model, pacing, action potential

## Abstract

**Introduction:**

Cell pacing is a fundamental procedure for generating action potentials (AP) in excitable tissues. Various pulse shapes have been proposed for this purpose, with the aim of either facilitating the achievement of the excitation threshold or minimizing energy delivery to the patient. This study seeks to identify the optimal pulse shape for each of these objectives.

**Methods:**

To determine the most effective pulse forms, we employed a mathematical model simulating nonlinear tissue responses to a range of pulse shapes.

**Results:**

Our results demonstrate that the rectangular pulse is optimal for reaching the excitation threshold, while the Gaussian pulse is superior in minimizing energy delivery. Other pulse shapes examined, including ramp-up, ramp-down, half-sine, and triangular (tent-like), fall between these two in terms of performance.

**Discussion:**

From a clinical perspective, the appropriate pulse shape should be selected based on the specific goal. For minimizing the pulse amplitude required to cross the excitation threshold, the rectangular pulse is recommended. In contrast, if reducing energy delivery to the patient is paramount, the Gaussian pulse is the preferred choice. In other scenarios, a judicious selection can be made based on the outcomes of our model and the clinical requirements.

## Introduction

In this study, we explore the optimal shapes of electric pulses, focusing on a set of commonly used pulse shapes for pacing applications in medicine and biophysics. Pacing is achieved either through a single pulse or a train of pulses, as seen in devices such as implantable cardioverter defibrillators (ICD) (Weiss et al., 2013). The goal of these devices is to use electrical stimulation to surpass a threshold (open voltage-gated channels) while minimizing energy delivered to the tissue to avoid adverse effects, such as pain. Two key objectives emerge in this context: threshold crossing and minimizing delivered energy, each potentially requiring different pulse properties.

For instance, in the treatment of atrial fibrillation (AF), the device may deliver a single pulse or a burst of pulses of varying shapes to the heart. For a single pulse of a given shape to terminate AF, its amplitude must be sufficient to cross the excitation threshold, and this amplitude varies depending on the pulse shape. Simultaneously, the energy imparted to the tissue by the threshold-crossing pulse differs with each shape. This study seeks to examine these differences.

As demonstrated in our recent research (Rabinovitch et al., 2023), *linear* pulse shape effectivity analyses often prove unreliable. The latter paper demonstrated that electric pacing by rectangular pulses for real (nonlinear) tissues reaches a minimum constant asymptotic energy deliverance to the patient when pulses’ durations get shorter and shorter. Short pulses with high amplitudes are used in High-output pacing in Cardiac Resynchronization Therapy to improve conduction velocity and cardiac output (Bavikati et al., 2012), to improve ventricular activation (Bonomini et al., 2017) and to achieve scar homogenization in ablation applications (Anderson et al., 2020). For too short pulses, the high pulse amplitudes can induce adverse effects for pacing purposes, namely, electroporation of cells, a process by which pores are created in the cell to either insert compounds (such as medications) into it or for “irreversible electroporation” to induce cell death (judicial cell death) (Napotnik, et al., 2021) by apoptosis, necrosis, or newer methods such as pyroptosis or necroptosis (Lv, et al., 2019). These effects are unwanted in pacing procedures. Electroporation has various applications including food decontamination, wound healing, and cancer treatment. It is reviewed extensively by Joshi et al. (2023) and is not investigated here.

Given that previous studies focused exclusively on rectangular pulses, the question arises as to whether other pulse shapes might offer reduced energy delivery and decreased patient discomfort compared to rectangular pulses. To address this, we conducted a series of calculations for various pulse shapes. As our analysis reveals, all pulse shapes exhibit similar behavior when pulse durations become very short (less than 5 milliseconds in heart tissue), converging towards a minimal energy plateau. However, our objective is to identify the most effective pulse shape for longer durations.

### The cardiac model

The Hodgkin-Huxley (HH) model, first proposed in (Hodgkin and Huxley, 1952), remains the foundational framework for understanding action potential (AP) generation in cells. It comprehensively accounts for the contributions of various ionic currents to the evolving membrane potential, making it highly representative of the underlying physiological processes. However, for specific applications such as cardiac electrophysiology, more specialized models like the Rogers-McCulloch or Fenton-Karma models are preferred due to their superior accuracy in reflecting the dynamics of cardiac tissue (e.g., Oliver et al., 2005; Biasi and Tognetti, 2021). When detailed ionic behavior is unnecessary and the primary focus is on the system’s nonlinear characteristics and overall dynamics, reduced models like the FitzHugh-Nagumo (FHN) system (FitzHugh, 1955; Nagumo et al., 1962) are frequently employed. The strengths and limitations of the FHN model are well documented (e.g., Yang et al., 2024) and will not be addressed here. For context, a description of the model for a rectangular pulse was provided by Rabinovitch et al. (2023). A brief overview is presented below for completeness.

In this study, we simulate the behavior of cardiac tissue using the nonlinear FHN system, governed by the following equations:

$$\frac{dv}{dt}=v\left(v-a\right)\left(1-v\right)-w+I\left(t\right)$$ (1)

$$\frac{dw}{dt}=\varepsilon \left(v-dw\right)$$

where *v* represents the action potential (AP) in the tissue, and *w* is an auxiliary variable that models inhibition. The parameters ε≪1, *a*, and *d,* are constants that align the FHN system with the Hodgkin-Huxley model. The term *I(t)* denotes the injected current, which takes on different pulse shapes depending on the pacing method.

**FIGURE 1:**
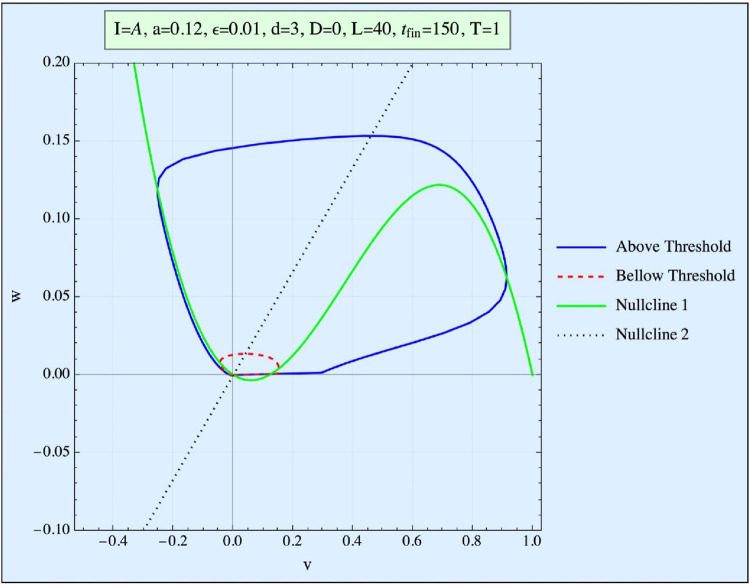
Phase space (w vs. v) of the FHN system. Presented are the curve obtained when the stimulation is below the threshold (red), and the trajectory obtained when it is above the threshold (blue). The nullclines represent the (v, w) curves of the first equation for no stimulation and no time development.

When this system is excitable (see Figure 1), the state of rest occurs when no external current is applied *I(t) = 0*, and the system stabilizes at a steady-state AP value (here, *v = 0*). When a single current pulse is introduced, the system’s response depends on the magnitude of the applied current. Only a negligible AP is generated if the current is below a critical threshold (red shift in Figure 1). However, if the current exceeds this threshold, a full AP pulse is produced, and the system returns to its resting state (blue trajectory in Figure 1).

In this model, the threshold concept is not an absolute value; rather, it is represented in phase space by a so-called Repeller line (R-line). The R-line, present in excitable systems, is a narrow region in phase space near the threshold, where trajectories undergo a smooth but abrupt directional change. This distinction between an R-line and a true threshold is explored in detail by Rabinovitch et al. (2016). Since biological systems typically exhibit thresholds of the R-type, we focus exclusively on this kind of threshold in our analysis.

The resting state of the model (with *v = w = 0*) represents the quiescent state of the system (simulating the ∼ −90 mV of the tissue), and the goal of the applied pulse(s) is to push the system beyond its threshold (or, more precisely, across the R-line), thereby triggering an AP pulse within the FHN system.

Time is expressed in time units (TUs), where 1 TU corresponds to approximately 3 milliseconds for the tissue (details provided below). The action potential (AP) voltage is measured in normalized units, with 0.3 of this unit (the threshold value) approximately equivalent to 20 mV in the tissue, making 1 unit roughly equal to 70 mV. Energy is reported in arbitrary units. These units are employed solely for the purpose of distinguishing between the different pulse shapes.

Note that the model treats a single location (0 dimensions). However, it does not mean that it refers to a single cardiac cell. Conversely, this location can refer to all contact points between the apparatus and the heart.

### Pulse shapes and energy delivery in the FHN model

**FIGURE 2:**
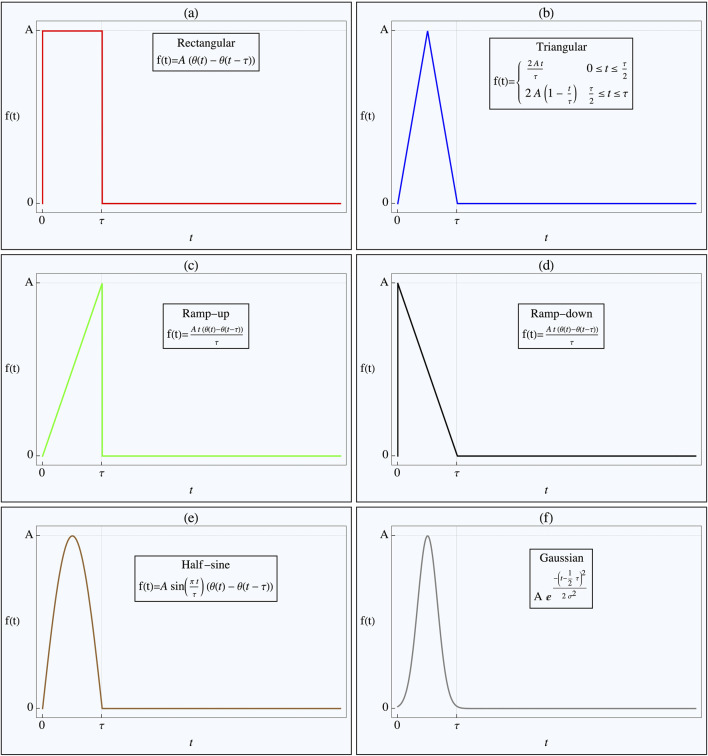
Pulse Shapes Applied to Nonlinear Tissue Simulation. The figure illustrates the various pulse shapes applied in the nonlinear tissue simulation, designed to determine the optimal shape for either achieving threshold crossing or minimizing energy delivery. Each pulse shape has the same duration, with its amplitude adjusted to ensure threshold crossing. The Heaviside step function, *H(t)*, defines the pulse onset. The specific pulse shapes shown in the different figure parts are as follows: **(a)** Rectangular pulse, **(b)** Triangular pulse, **(c)** Ramp-up pulse, **(d)** Ramp-down pulse, **(e)** Half-sine wave pulse, **(f)** Gaussian pulse.

Pulses of varying shapes, but identical durations τ (measured in time units (TU)), were applied to the FitzHugh-Nagumo (FHN) system for comparison (Figure 2). The typical pulse width of a pacemaker generally ranges from 0.4 to 1 millisecond (ms), though it may sometimes be as short as 0.2 ms or as long as 2 ms, depending on the specific device and the patient’s requirements. Pulse width refers to the duration of the electrical impulse delivered to stimulate the heart. To align our TU with real-time, we note that the pulse duration in our FHN model, defined as the period during which the pulse is positive, is ∼60 TU. Therefore, we calibrated our model to a typical pulse width of ∼200 milliseconds, such that

$$1\text{ TU}\approx 3_{\text{msec}}.$$

The pulse shapes tested included rectangular, rising triangular (ramp), decaying triangular, tent-like (double triangular), half-sinusoidal, and truncated Gaussian (with τ=6σ ). All pulses were of the same duration τ , and their amplitudes, $I\left(t\right)$ , were adjusted to ensure they reached the threshold (or more precisely, the R-line) by the end of the pulse duration. The energy delivered by each pulse to the tissue in order to reach the threshold was estimated using the following expression:

$$E=\int_{0}^{\tau }I\left(t\right)v\left(t\right)dt$$ (2)

where $I\left(t\right)$ represents the applied pulse current, and $v\left(t\right)$ is the response of the FHN system (Equation 1).

For short pulse durations and appropriately high pulse amplitudes $I\left(t\right)$ , where $I\left(t\right)$ ≫*v*, *w*, Equation 1 simplifies to: $\frac{dv}{dt}\approx I\left(t\right),$ for most of the pulse duration. Thus, the energy *E* can be approximated as:

$$E=\int_{0}^{\tau }v\left(\frac{dv}{dt}\right)dt=\frac{{v_{th}}^{2}}{2}$$ (3)

where $v_{th}$ is the threshold value of *v* reached at *t* = τ . This threshold is approximately located at the *w = 0* line in the phase space of Equation 1, making it nearly constant across all pulse shapes. Therefore, for sufficiently small τ , all threshold-reaching pulses, regardless of shape, will deliver approximately the same (minimal) energy.

As shown in previous studies (Rabinovitch et al., 2023), for the case where: *a* = 0.12, ε = 0.01, *d* = 3 in Equation 1, the threshold voltage $v_{th}=0$ .192. Thus, the minimum constant energy *Em* can be approximated as: *Em = 0.016*. This represents the energy delivered by all pulse shapes when τ is sufficiently small.

## Results

**FIGURE 3:**
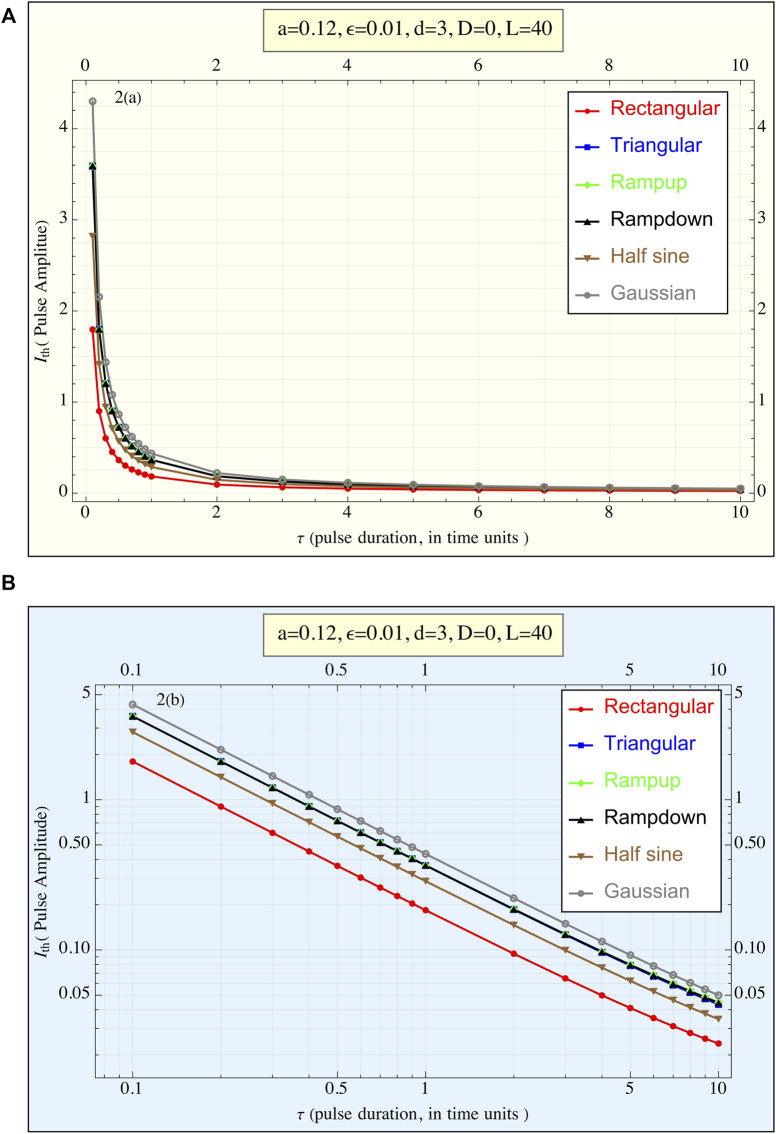
**(A)** The current amplitude required to reach the threshold ( $I_{th}$ ) is plotted against the pulse duration (τ) for all pulse shapes. This linear plot illustrates the relationship between pulse duration and the amplitude necessary to achieve the action potential threshold. Each pulse shape exhibits a hyperbolic-like curve, suggesting ( $I_{th}$ ) × *τ* ≈constant regardless of the pulse form, (*1TU* ≈ 3 msec for cardiac stimulations). **(B)** The current amplitude required to reach the threshold ( $I_{th}$ ) is plotted against the pulse duration *τ* for all pulse shapes, presented in a log-log plot (1TU ≈ 3 msec for cardiac stimulations). This representation highlights the differences in behavior between the pulse shapes more clearly than the linear plot. The rectangular pulse exhibits the lowest amplitude requirement across all durations, while the truncated Gaussian pulse requires the highest amplitude. Despite variations in amplitude, the overall trend of ( $I_{th}$ ) × *τ* ≈constant regardless consistent across different pulse shapes.

Using *a* = 0.12, ε = 0.01, *d* = 3 in Equation 1, Figure 3 presents the amplitudes required by various pulse shapes to reach the action potential (AP) threshold as a function of τ. In Figure 3A, plotted with linear coordinates, all curves display a hyperbolic form, suggesting that for each pulse shape, the relationship follows approximately $I_{th}\tau =\text{constant}$ . Where $I_{th}$ is the pulse amplitude necessary to reach the threshold at pulse duration τ.

In Figure 3B, the data is represented in a log-log plot, emphasizing the differences between the pulse shapes. It is evident that the *rectangular pulse requires the smallest amplitude to trigger* an AP, while the truncated *Gaussian pulse demands the highest amplitude*. This qualitative trend remains consistent across all *τ* values. Additionally, the three triangular-shaped pulses (ramp-up, ramp-down, and tent-like) exhibit nearly identical behavior. However, this performance trend reverses when considering delivered energy.

**FIGURE 4:**
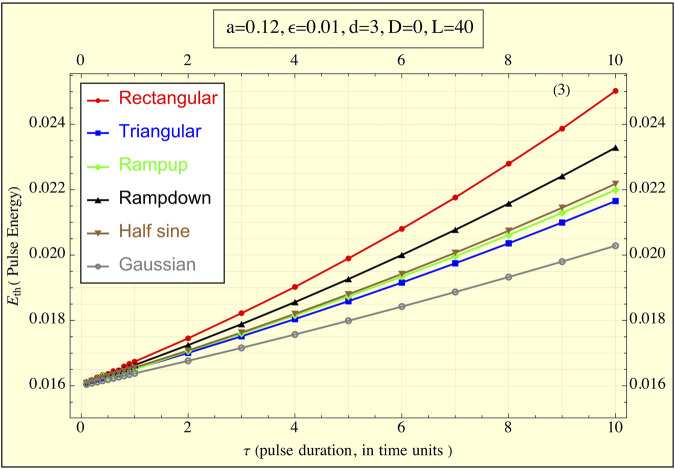
The energy delivered to the FitzHugh-Nagumo (FHN) system, simulating tissue, during the application of the pulse ( $\left.E_{th}\right)$ is plotted against pulse duration τ for all pulse shapes (1TU ≈ 3 msec for cardiac stimulations). The graph demonstrates how the delivered energy changes as a function of pulse duration. Notably, as τ decreases, the energies for all pulse shapes converge to the same minimal value, highlighting a universal behavior of the system for short pulse durations. Differences between pulse shapes are more pronounced at longer durations, with the rectangular pulse delivering the highest energy and the truncated Gaussian pulse the lowest.

Figure 4 illustrates the energy delivered by each pulse shape. Here, the rectangular pulse delivers the most energy, while the truncated Gaussian pulse delivers the least. Two further observations can be made: (1) all pulses converge to the same low energy value of approximately 0.016 for sufficiently small *τ*, consistent with earlier approximations, and (2) the ramp-up, triangular, and half-sine shapes deliver nearly identical amounts of energy across all *τ* values, whereas the ramp-down pulse requires more energy than these three.

## Discussion

Few clinical studies have evaluated the impact of pulse shapes on pacing outcomes. For instance, a pulse shape similar to our ramp-up configuration was tested using alternating magnetic fields on animals (Wieneke et al., 2013). Their findings indicated that “pacing thresholds for non-rectangular pulses were comparable to conventional pulse shapes in animal experiments.” Energy consumption at the pacing threshold ranged from 0.28 mJ to 5.36 mJ, with major determinants being distance and pacing threshold. Following our results here, we call on experimentalists to check these findings both for threshold crossing and energy delivered.

In cochlear devices, studies by Chen et al. (2023) and Navntoft et al. (2020) reported that upward ramp or triangular shapes “offered superior performance and caused less pain.” These findings suggest that clinicians should conduct more thorough investigations to understand how pulse shape affects the outcomes of different treatments. It is important to note that implantable cardioverter defibrillators (ICDs) or external devices typically do not generate the pulse shapes explored here. Instead, they often employ biphasic or triphasic pulses in addition to monophasic ones. Furthermore, due to the effects of capacitors, damped sine and exponential pulses are common in these devices (Laske et al., 2015). Such pulse shapes warrant exploration in future studies.

Additionally, since both threshold levels and pain tolerance can vary, patients with damaged tissues may exhibit different responses to pulses than those observed in this study. It is critical to specifically measure pulse-induced effects in these patients.

The superior performance of rectangular pulses in achieving threshold crossing can be attributed to their utilization of maximal amplitude throughout the entire pulse duration. Electrical conductivity in tissues varies with the intensity of the applied electric field (Lamberti et al., 2023), influencing the tissue’s response to pulse modulations. In our model, this effect is simulated by the FitzHugh-Nagumo (FHN) system’s response. According to Equation 2, to maximize electrical energy delivery to the tissue, the product of the pulse amplitude and the system’s response must be maximized throughout the pulse duration. This condition is met by the rectangular pulse shape, whereas other pulse forms, which vary in amplitude over time, achieve their peak currents during periods of lower system response.

Our approximation (Equation 3) of energy delivery behavior for very short and high pulses is reasonably accurate. The results indicate that reducing pulse duration while increasing amplitude decreases the energy delivered to the patient, but only up to a certain plateau. Thus, short pulses are effective, provided they are not excessively brief. High energy pulses, e.g., with durations longer than approximately 11 µs are preferable to avoid the risk of cell electroporation, a phenomenon associated with very high pulses (Casiola et al., 2017, Pakhomov and Pakhomova, 2020). They noted that electroporation occurs relatively quickly, while the stimulation process requires more time, suggesting that to prevent electroporation, pulse durations should exceed 11 µs.

In a model with diffusion, both v and w of Equation 1 are functions of t and x (if we treat a 1D case), and the v part of Equation 1 becomes:

$$\frac{dv\left(x,t\right)}{dt}=v\left(v-a\right)\left(1-v\right)-w+Dd^{2}x/dt^{2}+I\left(0,t\right)$$

To check our present results that don’t use diffusion, we ran several of our calculations (with different pulse shapes) containing diffusion (D = 10!) in one dimension. Results (not shown) show only extremely marginal differences both for the thresholds crossing and for the energies delivered. This insensitivity to diffusion is due to the relatively short duration of the pacing that does not permit spacial effects to interfere with the applied localized pulses. We maintain that these results will therefore also apply to a 3D case.

Recent technological advancements (Yuan et al., 2022) have enabled the development of pulse generators capable of producing pulses of various shapes, intensities, and durations. This progress allows for the judicious selection of pulse shapes, making it feasible to tailor devices to specific clinical needs.

## Conclusion

Our study shows strong numerical evidence of the following inferences:

1. Pulse Shape Impact: Various pulse shapes exhibit distinct behaviors when applied to a nonlinear system modeling real tissues, such as the heart.
2. Threshold Crossing: Rectangular pulses require the lowest amplitude to achieve threshold crossing, while Gaussian pulses necessitate the highest amplitude to reach the same threshold.
3. Energy Delivery: Conversely, in terms of energy delivered, rectangular pulses are the least favorable as they transmit the highest energy to the patient, whereas Gaussian pulses are preferable, delivering the least energy. Pulses with other shapes fall between these two extremes and can be selected based on specific requirements.
4. Clinical Implications: The energy delivered by pacing pulses is crucial for patient care, as excessively high energy can lead to pain or other adverse effects. Gaussian pulses are recommended for sensitive patients to minimize energy delivery, whereas rectangular pulses may be used for very short and high pulses to reduce the risk of electroporation.

We advocate for further clinical testing to determine the most appropriate pulse shapes for the various treatments.

## Data Availability

The raw data supporting the conclusions of this article will be made available by the authors, without undue reservation.
